# Discovery of benzethonium chloride as a potent STAT3 inhibitor for the treatment of HNSCC

**DOI:** 10.3389/fphar.2025.1569570

**Published:** 2025-04-02

**Authors:** Yuefeng Han, Shumin Li, Deshang Chen, Mingjie Zhang, Guoying Han, QianQian Xu, Xianming Ge, Mengli Wang, Yan Pan, Sien Ma, Beibei Xu, Mengmeng Deng, Bao Zhao

**Affiliations:** ^1^ Department of Otolaryngology, The First Affiliated Hospital of Bengbu Medical University, Bengbu, Anhui, China; ^2^ Institute of Health and Medicine, Hefei Comprehensive National Science Center, Hefei, Anhui, China; ^3^ School of Pharmacy, Center for Xin’an Medicine and Modernization of Traditional Chinese Medicine of IHM, Anhui University of Chinese Medicine, Hefei, Anhui, China; ^4^ Anhui Provincial Key Laboratory of Tumor Evolution and Intelligent Diagnosis and Treatment, Bengbu Medical University, Bengbu, Anhui, China; ^5^ School of Food and Biological Engineering, Hefei University of Technology, Hefei, Anhui, China; ^6^ Department of Clinical Laboratory, The First Affiliated Hospital of Bengbu Medical University, Bengbu, Anhui, China

**Keywords:** head and neck squamous cell carcinoma (HNSCC), apoptosis, benzethonium chloride (BZN), stat3, Mcl-1

## Abstract

Head and neck squamous cell carcinoma (HNSCC) is a prevalent malignancy with high mortality, and effective treatment strategies remain limited. This study investigated the effects of benzethonium chloride (BZN), an FDA-approved anti-infective agent, on HNSCC cell lines and its underlying mechanisms. BZN significantly inhibited the proliferation of HNSCC cell lines CAL27 and FaDu and induced apoptosis in both cell lines *in vitro*. In a subcutaneous tumor model, BZN markedly suppressed tumor growth in the mouse HNSCC cell line MOC1. Mechanistically, BZN may directly bind to the SH2 domain of STAT3, inhibit its dimerization, prevent the nuclear translocation of phosphorylated STAT3 (p-STAT3), downregulate the expression of the downstream protein MCL-1, and induce mitochondrial-mediated apoptosis in FaDu and CAL27 cells. These findings highlight BZN as a potential therapeutic agent for HNSCC, offering a novel approach to improve treatment outcomes in clinical settings.

## Introduction

Head and neck squamous cell carcinoma (HNSCC) accounts for approximately 90% of head and neck tumors and develops in the mucosal surfaces of the oral cavity, sinonasal cavity, larynx, and pharynx (Ruffin et al., 2023). HNSCC is the sixth most prevalent cancer globally, with 891,453 new cases and 458,107 deaths reported in 2022. In that year, HNSCC accounted for 4.4% of all new cancer cases and 4.7% of cancer-related deaths (Bray et al., 2024). HNSCC is commonly treated with surgical resection, chemotherapy, and radiotherapy (Johnson et al., 2020). For the treatment of locally advanced HNSCC, molecularly targeted agents, such as (the epidermal growth factor receptor (EGFR) inhibitor cetuximab) have shown modest success (Bonner et al., 2006). Immune checkpoint inhibitors (such as nivolumab and pembrolizumab) have been approved for treating recurrent or metastatic HNSCC (R/M HNSCC). However, the durable response rate for R/M HNSCC patients is less than 20% (Mell et al., 2024; Cramer et al., 2019). Therefore, it is imperative to seek novel therapeutic strategies for HNSCC.

The FDA-approved drug library provides a potential resource for developing antitumor agents. Benzethonium chloride (BZN) is an FDA-approved antiseptic and bactericide that has been identified as a broad-spectrum antitumor compound (Yip et al., 2006). Previous studies have demonstrated diverse mechanisms underlying BZN-induced antitumor activity in various cancers. For example, BZN inhibits the proliferation, migration, and invasion of osteosarcoma cells by repressing ERK1/2 signaling (Xia et al., 2023). In lung cancer, BZN may modulate the cell cycle, apoptosis, and epithelial–mesenchymal transition (EMT) by regulating the p53 signaling pathway or the phosphorylation of p38 (Tao et al., 2024; Huang et al., 2019). For hepatocellular carcinoma HCC treatment, BZN combined with endoxan led to significant increase of caspase-3-mediated apoptosis and remarkable decrease in diethylnitrosamine (DEN) induced primary HCC in rats (Abozaid et al., 2020). It has also been reported that BZN reduces proliferation by inducing ER stress in HNSCC (Rayess et al., 2018). However, the mechanism by which BZN provokes cell death remains elusive and needs to be clarified.

Signal transducer and activator of transcription (STAT) proteins include STAT1, STAT2, STAT3, STAT4, STAT5, and STAT6, which share 20%–50% sequence similarity (Li Y. J. et al., 2023). STAT3 is constitutively activated in multiple cancer types, such as HNSCC, breast cancer, lymphoma, melanoma, prostate cancer, and lung cancer, and plays an important role in tumorigenesis, progression, metastasis, and recurrence, resulting in poor clinical prognosis. In addition, the abnormal expression of STAT3 confers resistance to chemotherapy and targeted drug therapies (Wang et al., 2024). Hyperactivated STAT3 also plays a vital role in suppressing antitumor immune responses and inducing resistance to PD-1/PD-L1 antibody therapy (Zou et al., 2020). STAT3 contains an N-terminal domain (NTD), a coiled-coil domain (CCD), a DNA-binding domain (DBD), a linker domain (LD), a Src homology 2 domain (SH2), and a C-terminal transactivation domain (TAD) (Hu et al., 2024; Becker et al., 1998; Belo et al., 2019). Upon stimulation, the cytoplasmic inactive STAT3 is phosphorylated at Tyr705 and Ser727 residues in the TAD and becomes activated. Phosphorylation results in STAT3 dimerization and translocation from the cytoplasm to the nucleus, where STAT3 binds to target genes and facilitates transcription. To date, a variety of small-molecule STAT3 inhibitors have been reported, which typically bind to the CCD, DBD, or SH2 domain of STAT3. The SH2 domain is the most studied and advanced (Song et al., 2023). Accordingly, STAT3 has emerged as a potentially valuable target for cancer treatment.

In this study, we show that benzethonium chloride (BZN) has a strong antitumor effect on HNSCC both *in vitro* and *in vivo*. Mechanistically, we reveal that BZN may bind to the SH2 domain of STAT3, inhibiting STAT3 dimerization and translocation from the cytoplasm to the nucleus. This study highlights BZN as a potential drug for HNSCC therapy.

## Materials and methods

### Cell lines and drugs

The human pharyngeal squamous cell carcinoma cell line FaDu (ZQ0229, Shanghai Zhong Qiao Xin Zhou Biotechnology Co., Ltd.), the human laryngeal carcinoma cell line TU686 (Hefei All Things Biological Technology Co., Ltd.), TU177 cells (TU177 cells were kindly provided by Dr. Weiwei Liu, which were described previously (Liu et al., 2024) and the human tongue squamous cell carcinoma cell line CAL27 (CL-0265, Wuhan Pricella Biotechnology Co., Ltd.) were cultured in DMEM (Gibco, Thermo Fisher Scientific) supplemented with 10% fetal bovine serum (FBS) in a 5% CO_2_ incubator at 37°C. Benzethonium chloride (BZN) (B8879, Sigma-Aldrich) was dissolved in phosphate-buffered saline (PBS). The apoptosis inhibitor (Z-VAD-FMK, T7020, TargetMol), autophagy inhibitor (3-Methyladenine, 3-MA, T1879, TargetMol), ferroptosis inhibitor (Ferrostatin-1, Fer-1, T6500, TargetMol), and necroptosis inhibitor (Necrostatin-1, Nec-1, T1847, TargetMol) were dissolved in dimethyl sulfoxide (DMSO).

### CCK-8 assay

The effect of BZN on the proliferation of FaDu, TU177, TU686 and CAL27 cell lines were evaluated using the Cell Counting Kit (CCK-8, 40203ES80, Yeasen Biotechnology (Shanghai) Co., Ltd.). Briefly, 10,000 cells per well were seeded into a 96-well plate containing DMEM supplemented with 10% FBS. Cells were treated with different concentrations of BZN in triplicate. After treatment, 10 μL of CCK-8 reagent mixed with 90 μL of DMEM was added to each well, followed by incubation at 37°C for 2–4 h. Absorbance was measured at 450 nm using a microplate reader at 24, 48 and 72 h post-treatment. All experiments were performed in triplicate for reproducibility.

### Wound healing assay

A wound-healing assay was conducted to evaluate the effect of BZN on the migration of HNSCC cells. A total of 5 × 10^5^ cells per well were seeded into 6-well plates and cultured at 37°C in a 5% CO_2_ environment until reaching 100% confluence. A uniform scratch was created using a 200-μL pipette tip, and cells were washed with PBS. BZN was prepared at concentrations of 10 μM and 20 μM. The wound areas were observed and photographed at 0, 12, and 18 h-post-scratching. Scratch areas were quantified using ImageJ software. All experiments were performed in triplicate.

### Colony formation assay

Cells were seeded into 6-well plates at a density of 1,000 cells per well and treated overnight with BZN at concentrations of 0, 2, and 3 μM, followed by a 14-day incubation. Colonies were fixed with 4% paraformaldehyde (Yeasen Biotechnology, 40402ES50) for 15 min and stained with 0.1% crystal violet (Yeasen Biotechnology, 60505ES25) for 30 min at room temperature. Images of the colonies were captured and analyzed digitally. Colonies containing more than 50 cells were counted to calculate the survival fraction. All experiments were independently performed in triplicate.

### Migration assay

The migration ability of FaDu and CAL27 cells was assessed using 24-well Transwell chambers (6.5 mm diameter, 8-μm pore size, Corning Costar). Cells were suspended in serum-free medium at a concentration of 5 × 10^5^ cells/mL, and 100 μL of the suspension was added to the upper chamber. Complete medium (600 μL, supplemented with 10% FBS) was added to the lower chamber. After 4 h, the medium in the upper chamber was replaced with media containing BZN at 0, 10, and 20 μM. After 48-hour incubation, cells were fixed with 4% paraformaldehyde for 15 min and stained with 0.1% crystal violet for 30 min. Non-migrated cells were removed from the upper surface using cotton swabs, and migrated cells were counted in three random fields under a light microscope (40× magnification; Olympus Corporation). All experiments were performed in triplicate.

### Apoptosis assay

FaDu and CAL27 cells were cultured overnight in 12-well plates and treated with BZN (30 and 45 μM) or PBS (Control, Ctrl) for 48 h. Apoptosis rates were determined using the Annexin V-APC apoptosis detection kit (Yeasen Biotechnology, 40304ES50). Both floating and adherent cells were collected, washed three times with PBS, and stained with Annexin V and propidium iodide (PI) in binding buffer for 30 min in the dark. Apoptosis was analyzed using a NovoExpress flow cytometer (Agilent) and FlowJo software (v10). All experiments were performed in triplicate.

### CRISPR-Cas9-mediated gene knockout

The pLenti-CRISPR-V2 vector was used for CRISPR/Cas9-mediated gene knockout in CAL27 and FaDu cell lines. Briefly, lentivirus vector expressing *CASP3* gRNA (GAT​CGT​TGT​AGA​AGT​CTA​AC) was transfected together with psPAX 2and pMD2.G vectors into 293T package cells. After 72 h, virus supernatants were collected and filtrated with 0.2-μm filter. Target cells were infected twice and selected using 2 μg/mL puromycin for 3 days. Knockout effect was verified by Western blotting.

### Immunoprecipitation (IP) and Western blot analysis

For immunoprecipitation (IP), FaDu or CAL-27 cells were infected with lentivirus expressing STAT-Flag and STAT3-HA, treated with 20 μM BZN or left untreated for a duration of 48 h, and subsequently lysed in RIPA buffer supplemented with a protease inhibitor cocktail. Total protein extracts were incubated overnight at 4°C with Anti-DYKDDDDK Affinity Resin Easy (GenScript, L00907) under gentle agitation. After incubation, the samples were washed five times with cold RIPA buffer. Proteins were eluted from the beads by incubation with 30 μL of SDS sample buffer at 95°C for 10 min. For Western blot analysis, the proteins were separated by SDS-PAGE and transferred onto PVDF membranes. After blocking with 5% skim milk for 2 h, the membranes were incubated with primary antibodies overnight at 4°C, followed by three washes with TBST. Subsequently, the membranes were incubated with HRP-conjugated secondary antibodies for 2 h at room temperature. Protein detection was carried out using Clarity Western ECL substrate (Bio-Rad). Primary antibodies for Western blot included anti-β-actin (Sc-47778, Santa Cruz), Anti-Active caspase-3 Antibody (ET1602-47, Hanzhou Huaan Biotechnology, Co., Ltd.), BAX Antibody (B-9) (Sc-7480, Santa Cruz), BCL-2 Antibody (Sc-7382, Santa Cruz), MCL-1 Antibody (Sc-12756, Santa Cruz), XIAP Antibody (Sc-55550, Santa Cruz), SHP1 Rabbit mAb (R25713, Chengdu Zen-bioscience Co., Ltd.), STAT3 Rabbit mAb (R380907, Chengdu Zen-bioscience Co., Ltd.), Phospho-stat3(Tyr705) (D3A7)XP Rabbit mAb (9145, CST), Phospho-STAT3(Ser727) Rabbit mAb (R25804, Chengdu Zen-bioscience Co., Ltd.), p38(5A1) Mouse mAb (200782, Chengdu Zen-bioscience Co., Ltd.), Phospho-p38 MAPK (Thr180/Tyr182)Antibody (9211S, CST), JNK1 Rabbit mAb (R24778, Chengdu Zen-bioscience Co., Ltd.), Phospho-SAPK/JNK (Thr180/Tyr182) (81E11) Rabbit mAb (4668S, CST), ERK1/2 Rabbit mAb (R22685, Chengdu Zen-bioscience Co., Ltd.), Phospho-p44/42 MAPK (Erk1/2) Rabbit mAb (4370S, CST). NF-κB p65 Rabbit mAb (R25149, Chengdu Zen-bioscience Co., Ltd.), Phospho-NF-κB p65 Rabbit mAb (#3033, CST), IKB alpha Rabbit mAb (R380682, Chengdu Zen-bioscience Co., Ltd.), Phospho-IκBα (Ser32) (14D4) Rabbit mAb (#2589, CST), Rel B Rabbit mAb (R381877, Chengdu Zen-bioscience Co., Ltd.), AKT Rabbit mAb (R23412, Chengdu Zen-bioscience Co., Ltd.), Phospho-AKT(Thr308) Rabbit mAb (341790, CST) and Phospho-AKT(Ser473) Rabbit mAb (R381555, Chengdu Zen-bioscience Co., Ltd.). Goat anti-Rabbit IgG (H + L) Secondary Antibody, HRP (65-6120, Invitrogen) and Goat anti-Mouse IgG (H + L) Secondary Antibody, HRP (31431, Invitrogen) were used at 1:4000.

### Molecular docking

Molecular docking of BZN was performed using the MOE program (Vilar et al., 2008) based on the crystal structure of STAT3 (PDB entry: 6QHD) obtained from the Protein Data Bank. Molecular docking was carried out using the Dock module in MOE, with the scoring function ASE based on protein-ligand interaction energy. The docking results for BZN with the SH2 domain of STAT3 were obtained, where a lower binding energy indicates a more stable interaction between SH2 domain of STAT3 and BZN.

### Immunofluorescence assay

FaDu and CAL27 cells were seeded in 6-well plates at a density of 2 × 10^5^ cells per well and incubated overnight at 37°C in a 5% CO_2_ atmosphere. Cells were treated with 10 μM BZN or vehicle control for 48 h, washed, and fixed with 4% paraformaldehyde for 15 min. Permeabilization was carried out using 0.1% Triton X-100 in PBS, followed by blocking with 5% bovine serum albumin (BSA) for 1 h. Cells were then incubated with a STAT3 primary antibody overnight at 4°C. After washing with PBST, cells were incubated with Alexa Fluor™ 488-conjugated secondary antibody (Invitrogen, A-11008) at room temperature for 1 h, followed by DAPI staining (Thermo Scientific, 62248) for 15 min. Coverslips were mounted using an anti-quenching agent, and images were captured using an inverted fluorescence microscope.

### *In vivo* antitumor study

Animal experiments were approved by the Animal Experiment Ethics Committee of Bengbu Medical University (Approval No.: 078 [2023]) and conducted in compliance with institutional guidelines. To establish tumor xenografts, 3 × 10^6^ MOC1 cells were injected subcutaneously into the flanks of 6–8-week-old female *Rag1* ^*−/−*^ B6 mice. Once tumors reached a volume of approximately 100 mm^3^, mice were randomized into treatment and control groups. The treatment group received intraperitoneal injections of BZN (2.5 mg/kg) every 2 days, while the control group received equivalent volumes of PBS. Tumor size and body weight were measured every other day. Tumor volume was calculated using the formula: Volume = (Length × Width^2^)/2. Mice were euthanized 21 days after treatment initiation.

### Statistical analysis

Experimental data were analyzed using ImageJ (v1.8.0) and GraphPad Prism (v8.3.0). Results are presented as the mean ± SD. Statistical significance was determined using Student's t-tests, one-way ANOVA or two-way ANOVA where appropriate, with significance levels set at *p < 0.05, **p < 0.01, ***p < 0.001 and ****p < 0.0001. All experiments were performed in triplicate to ensure reproducibility.

## Results

### BZN inhibits HNSCC cell proliferation, migration and clonality

**FIGURE 1:**
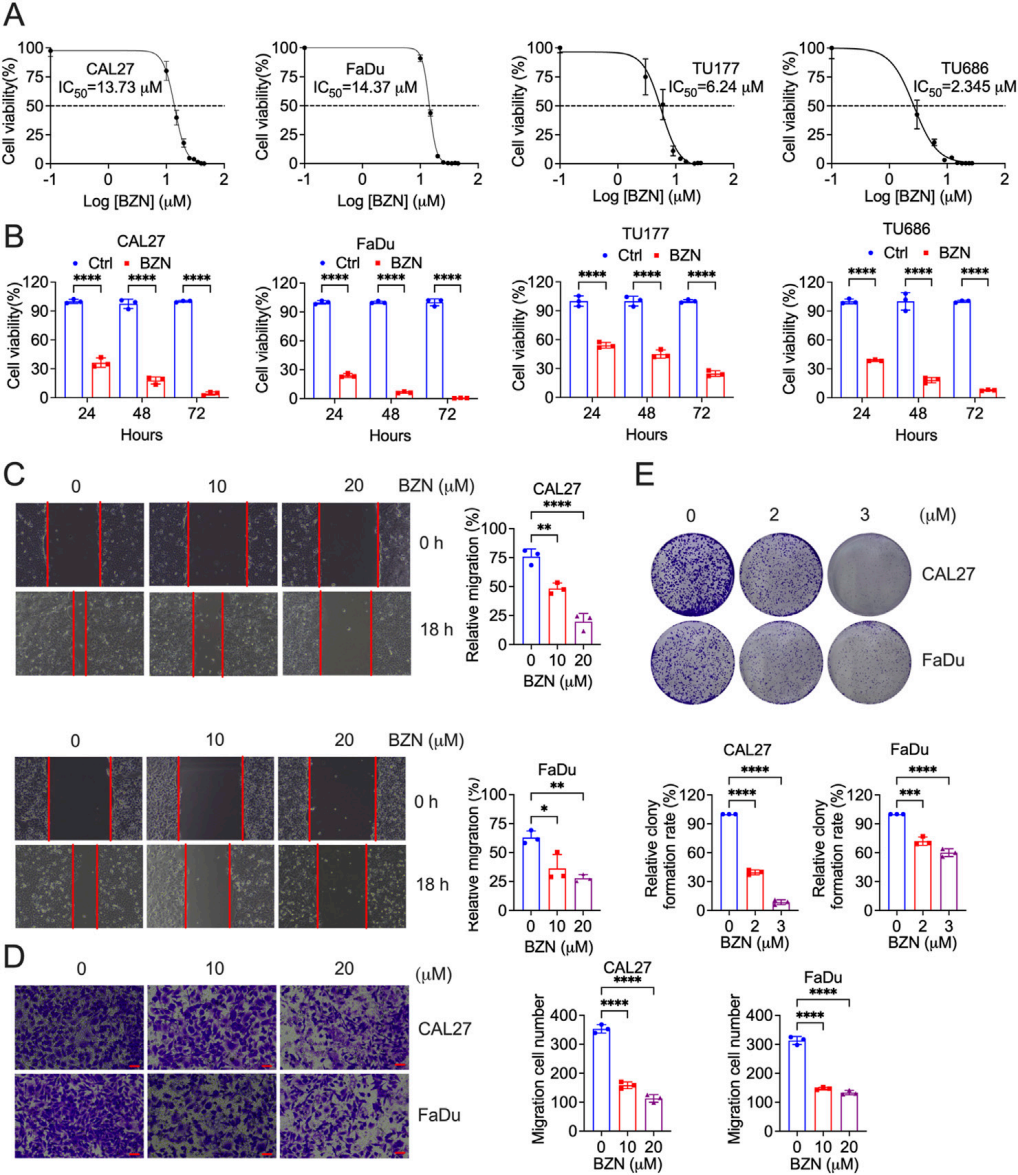
BZN inhibits HNSCC cell proliferation, migration, and clonality. **(A)** CAL27 and FaDu cells were treated with varying concentrations of BZN (10, 15, 20, 25, 30, 35, 40, and 45 μM), TU177 and TU686 cells were treated with different concentrations of BZN (3, 6, 9, 12, 15, 18, 21, 24 and 27 μM), and cell viability was assessed using the CCK-8 assay at 48 h **(B)** CAL27, FaDu, TU177 and TU686 cells were treated with or without BZN (20 μM for CAL27 and FaDu, 6 μM for TU177 and TU686), and cell viability was measured at 24, 48, and 72 h using the CCK-8 assay. **(C)** CAL27 and FaDu cells were seeded into 6-well plates to reach approximately 100% confluence. A uniform scratch was created in the cell monolayer using a 200-μL pipette tip, followed by treatment with 10 or 20 μM BZN, or DMSO as the control, for 24 h. Wound closure and cell migration were assessed. Representative images and bar graphs for each cell line (performed in triplicate) are shown. **(D)** Transwell migration assays were performed for CAL27 and FaDu cells treated with 0, 10, or 20 μM BZN. **(E)** Colony formation assays comparing CAL27 and FaDu cells treated with BZN (0, 2, and 3 μM). All data are presented as mean ± SD. **p ≤ 0.01, ***p ≤ 0.001, ****p ≤ 0.0001.

In order to evaluate the antitumor activity of BZN, HNSCC cell lines (CAL27, FaDu, TU177 and TU686) were treated with BZN at indicated concentration and time. Cell proliferation was assessed using the CCK-8 assay. As shown in Figures 1A, B, BZN significantly reduced the proliferation of CAL27 and FaDu cells in a dose- and time-dependent manner. The half-maximal inhibitory concentration (IC50) values at 48 h were 13.73 μM for CAL27, 14.37 μM for FaDu, 6.24 μM for TU177 and 2.345 μM for TU686 cells. To investigate the effect of BZN on cell migration, wound healing and Transwell migration assays were performed using CAL27 and FaDu cells. Figures 1C, D demonstrate that BZN treatment significantly impaired wound closure and cell migration, as quantified using ImageJ software. Additionally, the colony-forming ability of tumor cells was further investigated by treating CAL27 and FaDu cells with 2 or 3 μM BZN. Colony numbers were quantified and statistically analyzed. As depicted in Figure 1F, BZN markedly inhibited colony formation in both cell lines, confirming its potent anti-proliferative effects on HNSCC cells.

### BZN induces apoptosis and activates the mitochondria-mediated apoptosis signaling pathway

**FIGURE 2:**
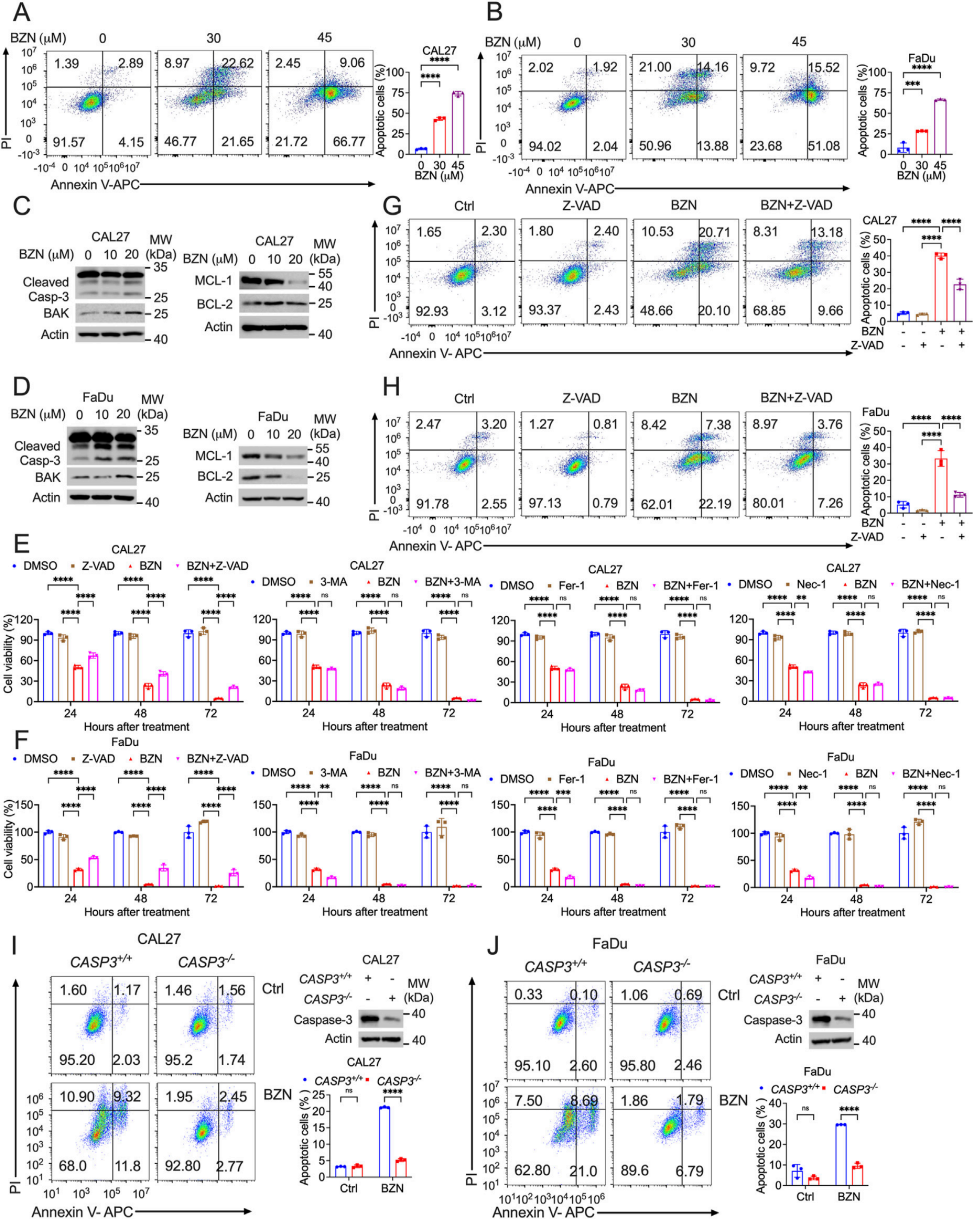
BZN induces apoptosis and activates mitochondrial-mediated apoptotic signaling. **(A, B)** Flow cytometry analysis of Annexin V-FITC/PI staining in CAL27 and FaDu cells treated with BZN (0, 30, and 45 μM) for 48 h demonstrates a dose-dependent increase in the apoptotic cell population. **(C, D)** Western blot analysis of CAL27 and FaDu cells treated with BZN (0, 10, and 20 μM) shows an increase in cleaved caspase-3 and BAK expression, alongside a decrease in BCL-2 and MCL-1 levels, indicating apoptosis induction. **(E, F)** CCK-8 assay showing the effect of apoptosis (Z-VAD, 20 μM), autophagy (3-MA, 5 mM), ferroptosis (Fer-1, 10 μM), and necroptosis (Nec-1, 50 μM) inhibitors on the cell death rate in CAL27 and FaDu cells treated with BZN for 48 h **(G, H)** The effect of Z-VAD on BZN-induced apoptosis in HNSCC cells, analyzed by Annexin V-FITC/PI staining. **(I, J)** Flow cytometry analysis of Annexin V-FITC/PI staining in *CASP3* ^*+/+*^ and *CASP3* ^*−/−*^ CAL27 or FaDu cells treated with BZN (30 μM) for 48 h. All data are presented as mean ± SD. ***p ≤ 0.001, ****p ≤ 0.0001.

To elucidate the mechanisms underlying BZN-induced cell death, Annexin V-FITC/PI staining was employed to assess the impact of BZN on tumor cell apoptosis. Flow cytometry analysis revealed a dose-dependent increase in apoptotic cells in CAL27 and FaDu cell lines treated with BZN (Figures 2A, B). Concurrently, Western blot analysis was conducted to detect the expression of apoptosis-related proteins. The results showed that the expression of cleaved caspase-3 and BAK was obviously increased, while the levels of BCL-2 and MCL-1 were remarkably decreased (Figures 2C, D).To further investigate the exact mechanism of BZN-induced cell death, HNSCC cells were treated with BZN and various cell death inhibitors, including Z-VAD (apoptosis inhibitor), 3-MA (autophagy inhibitor), Fer-1 (ferroptosis inhibitor), and Nec-1 (necroptosis inhibitor) for 24, 48 and 72 h, followed by cell viability assessment using CCK-8 assays. As shown in Figures 2E, F, only Z-VAD remarkably reversed BZN-induced cell death. Furthermore, to confirm that BZN can induce apoptosis in HNSCC cells, Annexin V-FITC/PI staining was performed. FACS analysis demonstrated that Z-VAD effectively rescued BZN-induced cell death (Figures 2G, H). Moreover, to investigate the critical role of apoptosis in BZN-induced cell death, *CASP3* were knockout by CRISPR-Cas9 system. As shown in Figures 2I, J, BZN effectively produced apoptosis in *CASP3* ^*+/+*^ FaDu and CAL27 cells but not *CASP3* ^*−/−*^ cells. Taken together, these results suggest that BZN inhibits HNSCC cell proliferation by inducing mitochondrial-mediated apoptosis.

### BZN induces anti-HNSCC activity through inhibiting phosphorylation of STAT3 at Y705

**FIGURE 3:**
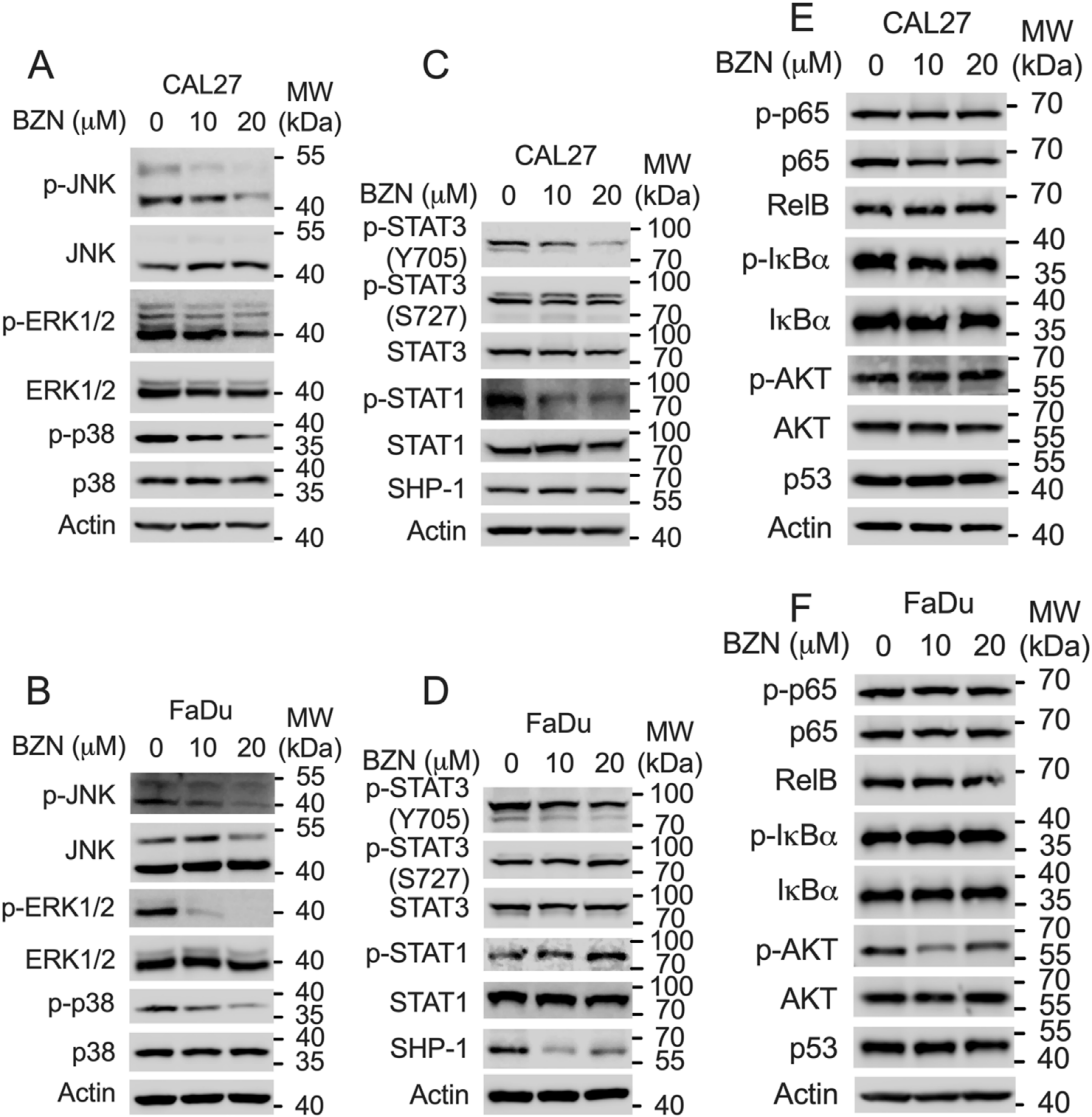
BZN inhibits STAT3 Y705 phosphorylation and the MAPK pathway. **(A, B)** Western blot analysis showing downregulation of phosphorylated ERK (p-ERK), p-p38, and p-JNK in CAL27 and FaDu cells treated with BZN (0, 10, and 20 μM). **(C, D)** Western blot analysis showing decreased phosphorylation of STAT3 at Tyr705 (p-TyrSTAT3), alongside expression changes in STAT1 and p-STAT1 in CAL27 and FaDu cells treated with BZN. **(E, F)** Expression levels of p53, p-p65, p65, p-IκBα, IκBα, Rel B, p-AKT, and AKT in CAL27 and FaDu cells treated with BZN (0, 10, and 20 μM), analyzed by Western blot.

To elucidate the molecular mechanisms underlying the inhibitory effects of BZN on the proliferation and metastasis of HNSCC, as well as its ability to induce apoptosis, we examined several key canonical signaling pathways by Western blot analysis. The RAS-MAPK pathway, which contributes to the growth and survival of HNSCC tumor cells, is frequently mutated in HNSCC tumors (Cancer, 2015). Previous studies have indicated that BZN activates the p38 signaling pathway without affecting ERK activation to inhibit lung cancer cell proliferation (Huang et al., 2019). However, our results demonstrated that BZN significantly decreased the phosphorylation of JNK, ERK, and p38 in HNSCC cells (Figures 3A, B). The STAT3 signaling pathway is abnormally activated in HNSCC and is associated with poor prognosis (Geiger et al., 2016). Thus, we explored the effect of BZN on STAT3 phosphorylation and found that BZN decreased the phosphorylation of Tyr705 (Y705) without affecting the phosphorylation of Ser727 (S727). Additionally, we observed that BZN had no effect on other STAT proteins, such as STAT1. Since SHP-1 plays a crucial role in the phosphorylation of STAT3, we assessed SHP-1 expression and found that BZN did not alter SHP-1 levels (Figures 3C, D). Furthermore, the PI3K-AKT-mTOR, NF-κB, and p53 pathways also play critical roles in HNSCC tumor development (Wang et al., 2017; Amit et al., 2020; Chung et al., 2010). Our data indicated that BZN did not regulate these pathways in HNSCC cell lines (Figures 3E, F). In conclusion, these findings suggest that BZN exerts anticancer effects by targeting STAT3 phosphorylation at the tyrosine 705 site.

### BZN disrupts STAT3 dimerization and nuclear translocation via direct interaction

**FIGURE 4:**
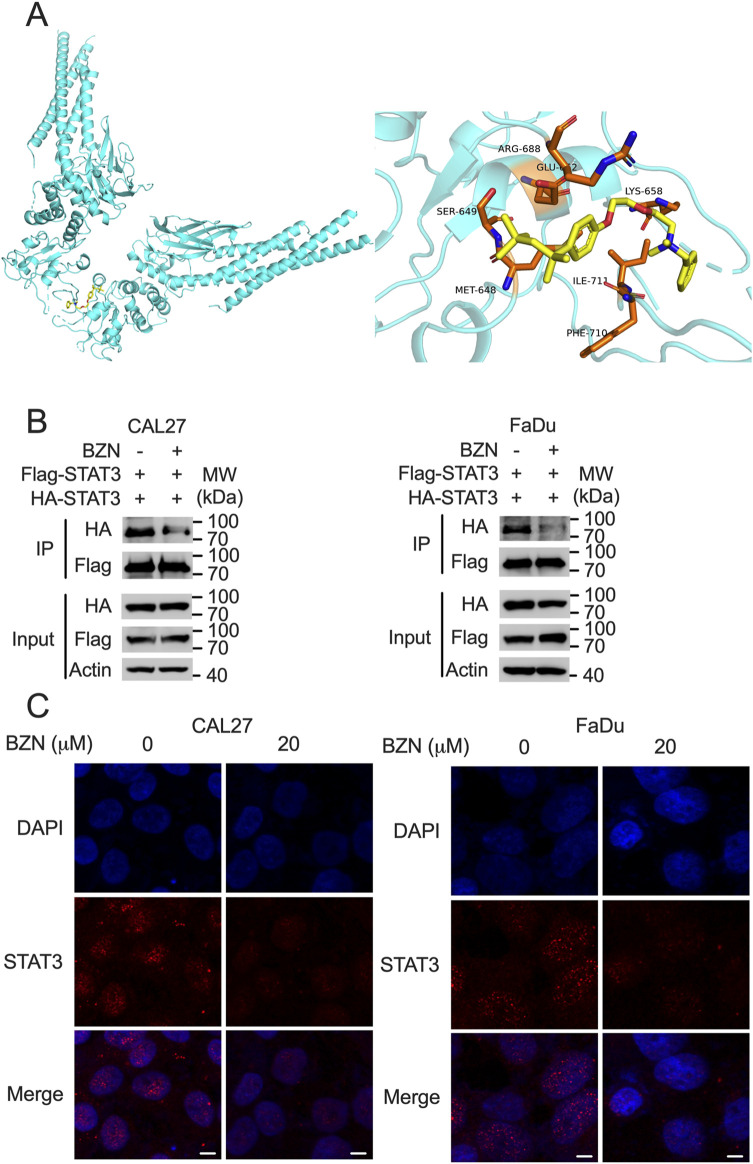
BZN directly interacts with STAT3, affecting its dimerization and nuclear translocation. **(A)** Computational docking model indicating the interaction of BZN with specific amino acids within the STAT3 SH2 domain. **(B)** Immunoprecipitation assay illustrating the effect of BZN on STAT3 dimerization. CAL27 and FaDu cells infected with lentivirus expressing Flag-STAT3 and HA-STAT3 were treated with 20 μM BZN or DMSO (control). Immunoprecipitation was performed using anti-Flag agarose, and the presence of HA-STAT3 in the precipitate was detected by Western blot. **(C)** Immunofluorescence analysis of STAT3 nuclear translocation following treatment with 10 μM BZN for 48 h. Cells were fixed and permeabilized, and STAT3 was visualized using a primary antibody and an Alexa Fluor 488-conjugated secondary antibody. Nuclei were counterstained with DAPI. Scale bar = 5 μm.

Because BZN decreased the phosphorylation of STAT3 without affecting the expression of SHP-1, we hypothesized that BZN inhibits STAT3 phosphorylation by directly binding to STAT3. The phosphorylation of the Tyr705 residue of STAT3 is essential for STAT3 homodimerization, nuclear translocation, and transcriptional activation (Chen et al., 2024). To evaluate whether BZN potentially interacts with the STAT3 SH2 domain, a computational docking simulation was performed using the STAT3 crystal structure [Protein Data Bank (PDB) entry 6QHD]. As shown in Figure 4A, BZN formed an extensive hydrogen bonding network with the side chains of the residues Arg688, Glu652, Ser649, and Lys658. Additionally, the residues Met648, Ile711, and Phe710 may interact with BZN through hydrophobic interactions. To investigate whether BZN inhibits STAT3 dimerization by directly binding to its SH2 domain, we performed immunoprecipitation (IP) experiments. CAL27 and FaDu cells were infected with lentivirus expressing Flag-STAT3 and HA-STAT3. Cells were treated with or without BZN for 24 h, then lysed and incubated with anti-Flag agarose beads for IP. The results showed that BZN effectively decreased the homodimerization of STAT3 (Figure 4B). Furthermore, to explore whether BZN binding to STAT3 reduces its nuclear translocation, we performed immunofluorescence experiments. The results indicated that BZN treatment significantly inhibited STAT3 nuclear translocation in HNSCC cell lines (Figure 4C). Taken together, these findings suggest that BZN interacts with STAT3, suppresses STAT3 dimerization, and inhibits its classical nuclear translocation, thereby restraining the expression of tumorigenesis-related genes.

### BZN potently suppresses HNSCC tumor growth *in vivo*

**FIGURE 5:**
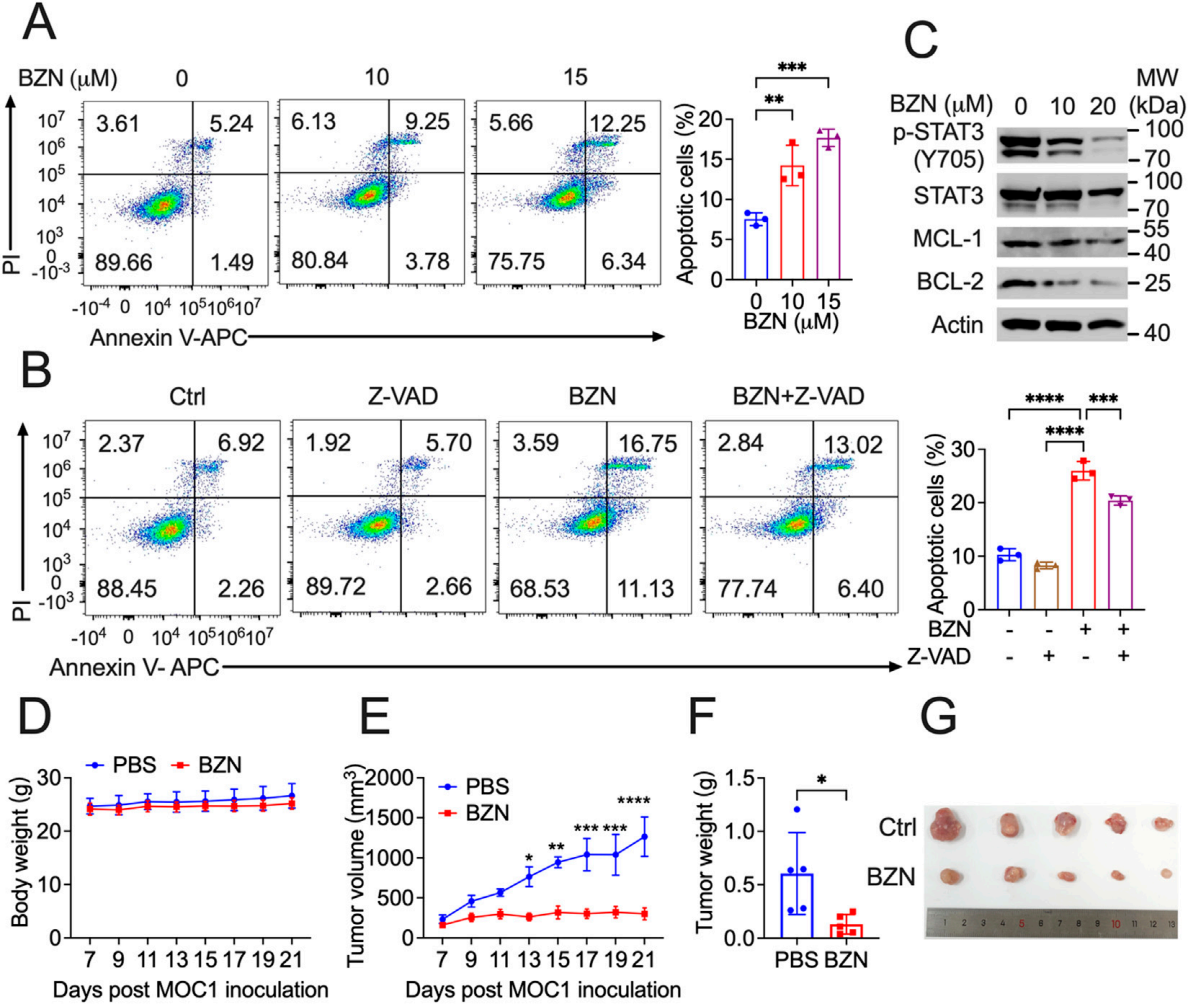
BZN suppresses HNSCC tumorigenesis *in vivo*. **(A, B)** Annexin V-FITC/PI staining demonstrating BZN-induced apoptosis in MOC1 cells, with and without the apoptosis inhibitor Z-VAD. **(C)** Western blot analysis showing downregulation of phosphorylated STAT3 (Y705) and BCL-2 protein in MOC1 cells treated with BZN (0, 10, and 20 μM). **(D, E)** *Rag1* ^*−/−*^ mice subcutaneously inoculated with MOC1 cells were treated with or without BZN. Mouse weight and tumor growth were monitored. **(F, G)** Tumor weight and representative tumor images were obtained on day 21 after inoculation. Images of tumors from the PBS-treated and BZN-treated groups are shown. All data are presented as mean ± SD. **p ≤ 0.01, ***p ≤ 0.001, ****p ≤ 0.0001.

Firstly, we confirmed that BZN could effectively induce apoptosis in mouse HNSCC MOC1 cells, and that Z-VAD significantly rescued this phenotype (Figures 5A, B). Furthermore, Western blot analysis showed that BZN markedly decreased the phosphorylation of STAT3 and the expression of BCL-2 and MCL-1 (Figure 5C). To assess the effects of BZN on HNSCC *in vivo*, *Rag1* ^*−/−*^ mice were subcutaneously injected with MOC1 cells. Once tumor volumes reached approximately 100 mm^3^, the mice were randomly assigned to different groups and intraperitoneally injected with BZN or vehicle control. BZN treatment significantly reduced the size of HNSCC MOC1 flank tumors without affecting the mice’s weight (Figures 5D–G). In summary, these results indicate that BZN suppresses the growth of HNSCC and holds potential for further pharmaceutical development.

## Discussion

Progress in drug development, robotic surgery, radiotherapy methods, biotechnology, and molecular diagnosis of HNSCC over the past 2 decades was expected to improve outcomes for HNSCC patients. However, outcomes for patients with recurrent/metastatic (R/M) HNSCC remain unchanged (Johnson et al., 2020). There is an urgent need to develop new approaches for R/M HNSCC. The development of novel therapeutics is a time-consuming and complex process. As a cost-effective alternative, repurposing existing compounds for anticancer applications has gained significant attention (Chong and Sullivan, 2007; Sachs et al., 2017). In this study, our data show that an FDA-approved compound, Benzethonium chloride (BZN), effectively inhibits HNSCC cell proliferation, migration, and colony formation. BZN also induces apoptosis in HNSCC cells, as confirmed by a Z-VAD rescue experiment. Furthermore, our results demonstrate that BZN significantly inhibits tumor growth *in vivo*. Mechanistic studies revealed that BZN markedly decreased the phosphorylation of STAT3 at Tyr705 (Y705) without affecting phosphorylation at Ser727 (S727). Additionally, BZN significantly inhibited the phosphorylation of JNK, ERK, and p38, which has not been reported in previous studies. Previous literature has shown that BZN activates ER stress and reduces proliferation in HNSCC SCC23 and HN12 cell lines, curbs FGL1 secretion, inhibits colon cancer liver metastasis, and negatively regulates MCU function, thereby delaying cell growth and migration in the triple-negative breast cancer cell line MDA-MB-231 (Rayess et al., 2018; Li J. J. et al., 2023; De Mario et al., 2021). Based on these studies, our research explored the novel signaling pathways underlying BZN-mediated antitumor activity in HNSCC. In addition, to support the clinical application of BZN for the treatment of HNSCC, a preclinical safety evaluation and an investigator-initiated clinical trial should be conducted in the future.

STAT3, a member of the STAT transcription factor family, facilitates tumor progression by regulating genes associated with cell metabolism, survival, proliferation, metastasis, and immune evasion (Li Y. J. et al., 2023; Yu et al., 2014). STAT3 is hyperactivated in various tumors, including HNSCC (Wang et al., 2024). To date, numerous STAT3 inhibitors, including peptides, oligonucleotides, and small molecular compounds, have been reported and are currently being investigated in preclinical or clinical studies (Wang et al., 2024; Chen et al., 2024; Huynh et al., 2019; Jin et al., 2022). In this study, we present BZN as a novel inhibitor of STAT3. We found that BZN inhibits phosphorylation of STAT3 at Y705, but not at S727, and has no effect on the expression of SHP-1 or the activation of STAT1. We hypothesized that BZN may bind to the SH2 domain of STAT3, which was confirmed through computational modeling. Furthermore, we confirmed that BZN could reduce STAT3 homodimerization, nuclear translocation, and decrease the expression of MCL-1 and BCL-2. Since STAT3 plays a critical role in inhibiting the expression of key immune activation regulators and enhancing the production of immunosuppressive factors (Zou et al., 2020), future studies should explore the impact of BZN on the tumor microenvironment. Previous studies have indicated that MAPK pathway molecules, such as ERK, p38, and JNK, can activate S727 phosphorylation of STAT3 (Chen et al., 2024; Huynh et al., 2019). However, in our study, we showed that BZN significantly decreases the phosphorylation of ERK, p38, and JNK without affecting STAT3 S727 phosphorylation, which warrants further investigation.

In conclusion, this study reveals a novel small-molecule STAT3 inhibitor, BZN, which inhibits STAT3 phosphorylation at Y705, homodimerization, and nuclear translocation. BZN potently inhibits HNSCC cell proliferation, migration, and clonality and induces mitochondrial-mediated apoptosis *in vitro*, while reducing tumor growth *in vivo*. Mechanistic studies show that BZN inhibits STAT3 Y705 phosphorylation, homodimerization, and nuclear translocation. Therefore, this study not only evaluates the potential of BZN as an anti-tumor drug or drug lead for HNSCC but also offers new insights into the development of novel STAT3 inhibitors.

## Data Availability

The raw data supporting the conclusions of this article will be made available by the authors, without undue reservation.
